# Association of transglutaminase-2 with contrast-induced acute kidney injury in Chinese patients with acute coronary syndrome: a prospective cohort study

**DOI:** 10.1080/07853890.2026.2712061

**Published:** 2026-08-10

**Authors:** Ruixue Yao, Jie Zhang, Wenkang Zhang, Yuhan Qin, Yong Qiao, Dong Wang, Gaoliang Yan, Chengchun Tang

**Affiliations:** a School of Medicine, Southeast University, Nanjing, China; b Department of Cardiology, Zhongda Hospital Affiliated to Southeast University, Nanjing, China; c Jinling Hospital, Affiliated Hospital of Medical School, Nanjing University, Nanjing, China

**Keywords:** Transglutaminase-2, contrast-induced acute kidney injury, acute coronary syndrome, coronary angiography, percutaneous coronary intervention

## Abstract

**Background:**

To investigate the dynamic changes in serum and urinary Transglutaminase-2 (TG2) levels in acute coronary syndrome (ACS) patients and their association with contrast-induced acute kidney injury (CI-AKI), thereby providing novel clinical evidence for risk prediction of CI-AKI.

**Methods:**

This single-center prospective cohort study consecutively enrolled ACS patients hospitalized in the Zhongda Hospital Affiliated to Southeast University, who underwent coronary angiography (CAG) between December 2023 and December 2024. Serum and urinary TG2 levels were measured before and after CAG using enzyme-linked immunosorbent assay. Multivariable logistic regression was performed to identify independent risk factors for CI-AKI, and a predictive model was constructed.

**Results:**

A total of 550 ACS patients were included, of whom 59 (10.7%) developed CI-AKI. Lasso regression analysis further confirmed that the differences between preoperative and postoperative serum TG2 (ΔsTG2) and urinary TG2 (ΔuTG2) were independent risk factors for CI-AKI (both *p* < 0.001). The logistic regression prediction model incorporating ΔsTG2, ΔuTG2, and other risk factors achieved area under the curve values of 0.870 (95% confidence interval [CI]: 0.818–0.921) in the training set and 0.818 (95% CI: 0.741–0.895) in the test set, indicating good predictive performance.

**Conclusion:**

In ACS patients undergoing CAG, elevated serum and urinary TG2 levels and greater peri-procedural changes in these levels are closely associated with the occurrence of CI-AKI. Detecting TG2 levels, particularly their dynamic changes, may serve as an effective biomarker for predicting the risk of CI-AKI.

## Introduction

Contrast-induced acute kidney injury (CI-AKI) is defined as acute renal impairment occurring within 48–72 h after intra-arterial administration of iodinated contrast media, in the absence of other identifiable causes of acute kidney injury [1,2]. While CI-AKI can occur following both intravenous and intra-arterial contrast administration, the incidence is substantially higher with intra-arterial administration [3], which explains why coronary angiography (CAG) and percutaneous coronary intervention (PCI) are associated with the highest incidence among all contrast-assisted diagnostic and therapeutic procedures [4]. CI-AKI is the third most common cause of hospital-acquired acute kidney injury, following only renal hypoperfusion and drug-induced kidney injury [1,2]. CI-AKI predominantly affects elderly patients with underlying conditions such as diabetes mellitus, hypertension, chronic kidney disease (CKD), and heart failure. Its occurrence is associated with significantly prolonged hospital stays, increased incidence of adverse cardiovascular events, and elevated long-term mortality, imposing a substantial burden on clinical practice [5]. The incidence of CI-AKI varies considerably across populations: it is approximately 0.6%–2.3% in the general population undergoing contrast-enhanced procedures [5], whereas patients with acute coronary syndrome (ACS), who often have complex underlying vascular pathologies and reduced renal functional reserve, exhibit a substantially higher incidence after CAG or PCI, with some studies reporting rates as high as 50% [6]. Meta-analyses have confirmed that intra-arterial contrast administration confers a significantly higher risk of CI-AKI than intravenous administration [3], which explains why CAG and PCI are associated with the highest incidence of CI-AKI among all contrast-assisted diagnostic and therapeutic procedures [4]. To date, the pathogenesis of CI-AKI remains incompletely understood. Current research implicates multiple pathophysiological processes, including medullary ischemia and hypoxia, direct contrast-induced nephrotoxicity, oxidative stress, tubular cell apoptosis, and inflammatory infiltration [7]. Further investigation into key regulatory molecules is warranted to identify novel targets for prevention and treatment.

Transglutaminases (TGases) comprise a family of eight enzymes with protein cross-linking activity, broadly involved in various physiological processes such as cell proliferation, differentiation, apoptosis, and matrix remodeling. They are also closely associated with the pathogenesis of diverse diseases, including cancer [8], organ inflammation [9–11], neurological disorders [12], and autoimmune diseases [13]. Previous studies have shown that urinary TGase levels are significantly elevated in a mouse model of cisplatin-induced AKI, suggesting that injured renal tubular cells may upregulate TGase expression and release it into the urine following acute renal insult [14], indicating a potential role for TGases in the pathophysiology of AKI.

Transglutaminase-2 (TG2) is the most widely distributed and functionally complex member of the TGase family. First isolated and characterized in 1957, TG2 is expressed in various subcellular compartments across multiple cell types and exhibits diverse enzymatic activities, including transamidase, protein disulfide isomerase, and GTP/ATPase activities [15,16]. Multiple studies have established a close association between TG2 and various kidney diseases. Da Silva Lodge et al. measured urinary TG2 levels in patients with CKD and in a rat model of CKD, finding that urinary TG2 levels were closely correlated with disease progression and could serve as a potential biomarker for predicting CKD progression [17]. Moon et al. demonstrated that treatment with the TG2 inhibitor cystamine significantly ameliorated renal tissue injury and fibrosis by suppressing TG2-mediated renal cell apoptosis, inflammatory responses, and extracellular matrix accumulation, further underscoring the pathogenic role of TG2 in CKD [18]. A domestic study on children with Henoch–Schönlein purpura reported that urinary TG2 levels in patients with Henoch–Schönlein purpura nephritis were negatively correlated with 24-hour urinary protein excretion and could serve as an effective biomarker for early diagnosis and prediction of purpura-related renal injury [19]. However, to date, no studies have investigated the expression changes, mechanisms of action, or predictive value of TG2 in CI-AKI.

Therefore, TG2 may play a critical role in the pathogenesis of CI-AKI by participating in pathological processes such as inflammation, renal tissue hypoxia, and renal tubular cell apoptosis. This study aims to investigate the association between dynamic changes in serum and urinary TG2 levels before and after CAG and the occurrence of CI-AKI in ACS patients, and to construct a predictive model incorporating TG2 for CI-AKI, thereby providing new insights and clinical evidence for risk assessment of CI-AKI.

## Methods

### Study design and participants

This study was a single-center prospective cohort study conducted in strict accordance with the Strengthening the Reporting of Observational Studies in Epidemiology (STROBE) statement [20]. The study procedures included patient enrollment, collection of baseline data, CAG/PCI procedures, postoperative follow-up, and outcome assessment. Patients were divided into the CI-AKI group and the non-CI-AKI group based on the occurrence of CI-AKI after the procedure.

Patients with ACS who were hospitalized in the Zhongda Hospital Affiliated to Southeast University, and underwent CAG between December 2023 and December 2024 were consecutively enrolled. Inclusion criteria were as follows: (1) meeting the diagnostic criteria for ACS according to the Chinese Guidelines for the Diagnosis and Treatment of Acute Coronary Syndrome (2023 Edition) and scheduled to undergo CAG; (2) aged 18–90 years; and (3) voluntary participation in the study with written informed consent. Exclusion criteria were as follows: (1) age <18 years or >90 years; (2) history of allergy to iodinated contrast media; (3) use of nephrotoxic drugs (e.g. aminoglycoside antibiotics, nonsteroidal anti-inflammatory drugs) within the previous 2 weeks or pre-existing acute renal insufficiency; (4) exposure to any contrast-enhanced examination (including computed tomography, magnetic resonance imaging, etc.) within the previous 2 weeks; (5) admission for emergency PCI (defined as patients with acute STEMI presenting within 12 h of symptom onset, or very high-risk NSTEMI patients. Patients with STEMI presenting beyond the recommended reperfusion time window (i.e. >12 h from symptom onset), or those who received thrombolytic therapy and underwent subsequent delayed catheterization, were eligible for inclusion provided that baseline data collection and hydration were feasible; (6) severe chronic renal insufficiency [CKD stage 4–5, estimated glomerular filtration rate (eGFR) <30 mL/min/1.73 m^2^], patients undergoing maintenance hemodialysis, or those with a history of kidney transplantation; (7) hemodynamic instability, or intraoperative requirement for life-support devices such as intra-aortic balloon pump or extracorporeal membrane oxygenation; (8) presence of malignant tumors, hyperthyroidism or hypothyroidism, severe hepatic insufficiency (Child-Pugh class C), or severe infections (e.g. sepsis); and (9) missing >20% of key clinical or laboratory data, precluding statistical analysis.

This study was approved by the Ethics Committee of Zhongda Hospital Affiliated to Southeast University (approval No. 2022ZDSYLL182-P01). All patients provided written informed consent before enrollment, and the study was conducted in accordance with the principles of the Declaration of Helsinki.

### Data collection

Baseline clinical data were collected, including general demographic characteristics (age, sex, body mass index [BMI]), and smoking status. Smoker was defined as current active cigarette smoking at the time of admission; former smokers (quit >6 months) and passive smokers were categorized as non-smokers. Medical history included hypertension (systolic blood pressure ≥140 mmHg and/or diastolic blood pressure ≥90 mmHg on at least two occasions, or current antihypertensive medication use), diabetes mellitus (DM) (fasting plasma glucose ≥7.0 mmol/L, HbA1c ≥6.5%, 2-hour glucose ≥11.1 mmol/L during OGTT, or current antidiabetic medication use) [reference], and CKD (eGFR <60 mL/min/1.73 m^2^ for ≥3 months or evidence of kidney damage for ≥3 months). Medication history (antiplatelet agents, antihypertensive drugs, antidiabetic agents, statins) was recorded based on medications prescribed at the time of admission. Fasting venous blood and morning urine specimens were obtained on the morning after admission (preoperative) and within 72 h postoperatively. Serum was separated after centrifugation (3000 r/min, 10 min, 4 °C), and urine supernatant was collected after centrifugation (1500 r/min, 5 min, 4 °C). All specimens were stored at −80 °C for subsequent batch testing. Clinical laboratory tests included glycated hemoglobin (HbA1c), triglycerides (TG), total cholesterol (TC), low-density lipoprotein cholesterol (LDL-C), high-density lipoprotein cholesterol (HDL-C), serum creatinine (sCr), blood urea nitrogen (BUN), eGFR (calculated using the CKD-EPI equation), and uric acid (UA). All tests were performed by the Department of Clinical Laboratory, Zhongda Hospital Affiliated with Southeast University, following standardized procedures. In addition, data on CAG parameters (puncture site, contrast agent type and volume), PCI details (stent implantation status, number of stents), and postoperative hydration status were recorded.

### Serum and urinary TG2 level measurement

Serum and urinary TG2 levels were measured using enzyme-linked immunosorbent assay (ELISA) with a human transglutaminase-2 ELISA kit (Jiangsu Meimian Industrial Co., Ltd., Cat. No. MM-1234H2). All procedures were performed strictly in accordance with the manufacturer’s instructions. The detection wavelength was 450 nm, and TG2 concentrations were calculated using a standard curve. All specimens were tested in duplicate, and the difference in absorbance between duplicates was considered acceptable if <10%; the mean value was taken as the final result.

### Contrast agent administration and hydration protocol

All patients undergoing CAG received a loading dose of antiplatelet therapy preoperatively: 300 mg of aspirin chewed and swallowed, combined with 300 mg of clopidogrel bisulfate or 180 mg of ticagrelor orally. CAG procedures were performed by experienced cardiovascular interventional physicians in the catheterization laboratory at Zhongda Hospital Affiliated with Southeast University, using the modified Seldinger technique with radial or femoral artery puncture. PCI was performed based on the severity of coronary lesions. Either an iso-osmolar contrast agent (iodixanol) or a low-osmolar contrast agent (iopromide) was selected, with the volume adjusted according to procedural requirements to avoid excessive use. The standardized hydration protocol followed the regimen recommended by the 2018 ESUR guidelines [21]: intravenous infusion of 0.9% saline was prescribed to be initiated 3–4 h before contrast injection and continued until 4–6 h after injection, at a rate of 1 mL·kg^−1^·h^−1^. For patients with concomitant heart failure (NYHA class III-IV or ejection fraction <40%), the infusion rate was appropriately adjusted to 0.5 mL·kg^−1^·h^−1^ to avoid exacerbating cardiac burden. While hydration was prescribed for all patients, actual completion of the full protocol was not always feasible due to clinical factors including (1) acute heart failure exacerbation requiring diuretic therapy; (2) procedural complications or delays; (3) patient intolerance (e.g. dyspnea, fluid overload); (4) early discharge before completion of postoperative hydration; or (5) clinical deterioration requiring termination of hydration. Therefore, for each patient, we recorded hydration completion status (yes/no), defined as successful completion of both the preoperative and postoperative hydration components as prescribed. The hydration completion rate was calculated as (number of patients who completed hydration/total number of patients) × 100%.

### Outcome definition

The primary outcome of this study was CI-AKI, defined according to the Kidney Disease: Improving Global Outcomes (KDIGO) criteria recommended by the Contrast Media Safety Committee of the European Society of Urogenital Radiology (ESUR) in 2018: an increase in sCr of >26.5 μmol/L (0.3 mg/dL) from baseline, or an increase to 1.5–1.9 times the baseline value, within 48–72 h after intravascular administration of iodinated contrast medium (in this study, intra-arterial administration during CAG), after excluding other causes of acute kidney injury (such as renal hypoperfusion, drug-induced kidney injury, infection, etc.) [21].

### Statistical analysis

All statistical analyses were performed using RStudio software (version 4.2.3). The required sample size was estimated based on the anticipated incidence of CI-AKI (10%) and the expected effect size of ΔsTG2 (OR = 2.0 for ΔsTG2 above vs. below the median). With α = 0.05 (two-sided) and power = 80%, the minimum sample size was calculated to be 450 patients. Accounting for a 10% rate of incomplete data, we aimed to enroll at least 500 patients. The final cohort of 550 patients exceeded this requirement. We acknowledge that with 59 CI-AKI events and 8 predictors in the final model, the events per variable (EPV) ratio was 7.4, slightly below the recommended 10 EPV rule [22]. To address this, we employed Lasso regression with cross-validation for variable selection and performed internal validation using a train/test split to reduce overfitting risk. Two-sided tests were used, and a *p* value <0.05 was considered statistically significant. Categorical variables were presented as counts (percentages), and group comparisons were performed using the chi-square test; Fisher’s exact test was used when the expected frequency was <5. Continuous variables were first tested for normality using the Shapiro–Wilk test. Normally distributed data were presented as mean ± standard deviation and compared using the independent samples t-test. Non-normally distributed data were presented as median (interquartile range) and compared using the Mann–Whitney U rank-sum test.

Linear regression models were used to assess the correlation between TG2 levels and various clinical indicators. The Boruta algorithm was employed to initially screen for characteristic variables influencing the occurrence of CI-AKI. Subsequently, Lasso regression was used to further select key variables and eliminate redundant ones. Multivariable logistic regression was performed to identify independent risk factors for CI-AKI. Multicollinearity among predictor variables was assessed using Variance Inflation Factor (VIF), with a VIF >5 considered indicative of concerning collinearity. The predictive performance of the logistic regression model constructed based on the Lasso-selected variables was evaluated using receiver operating characteristic (ROC) curves, and the area under the curve (AUC), optimal cut-off value, sensitivity, and specificity were calculated. Calibration curves were plotted to assess model goodness-of-fit, and the Hosmer–Lemeshow test was used to validate calibration performance. A nomogram prediction model was constructed based on the regression coefficients from the Lasso analysis to facilitate individualized risk assessment for CI-AKI. To evaluate the clinical utility of the prediction model, decision curve analysis (DCA) was performed. The net benefit of the model was calculated across a range of threshold probabilities (0 to 0.5) and compared with two reference strategies: intervening on all patients (‘treat all’) and intervening on no patients (‘treat none’). A model is considered clinically useful if it provides higher net benefit than both reference strategies across a reasonable range of threshold probabilities.

## Results

### Baseline characteristics

**Figure 1.**
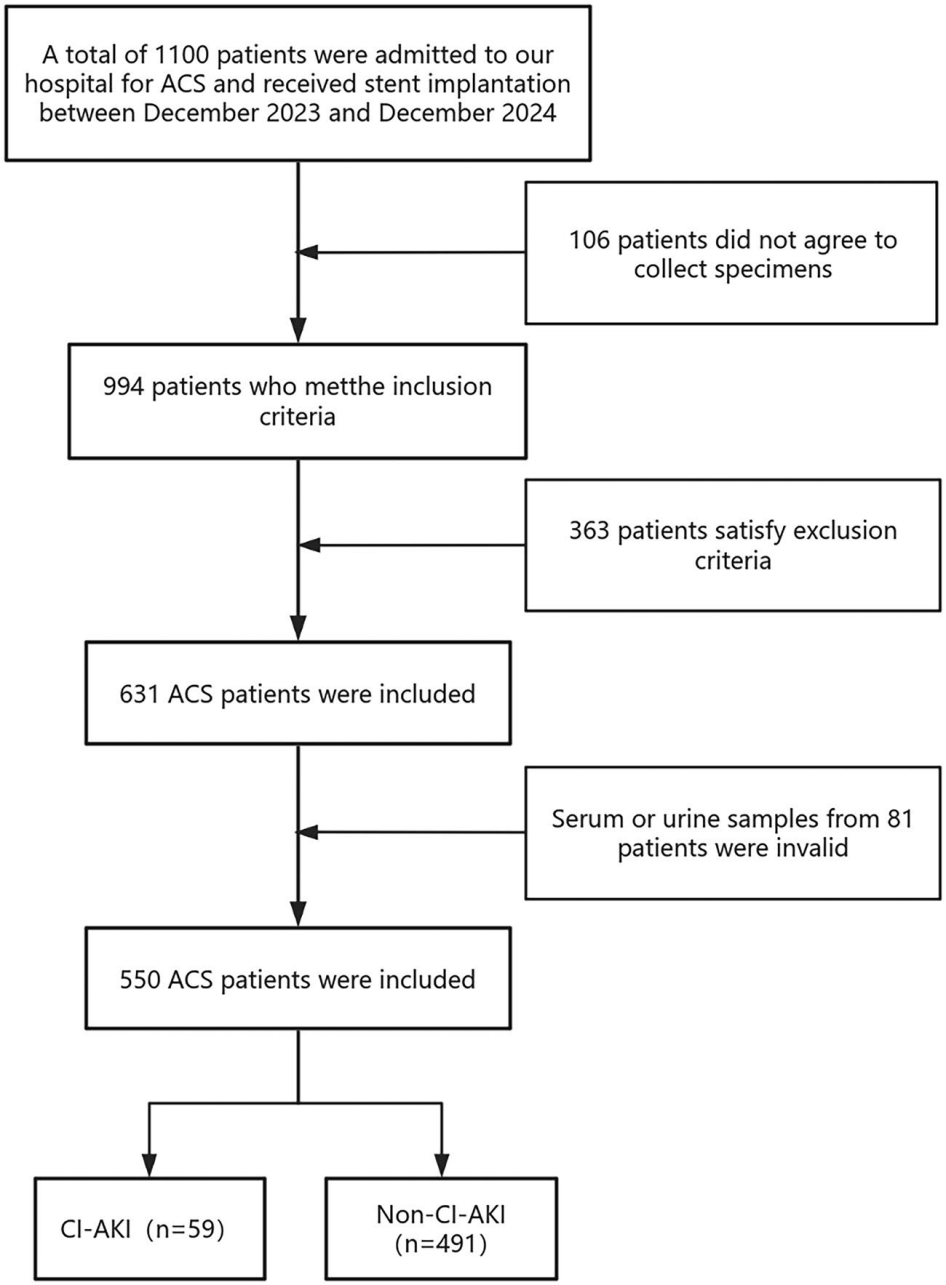
Study Flowchart: Patient Screening, Enrollment, and Grouping. Flowchart showing the screening, enrollment, and grouping of patients with acute coronary syndrome (ACS) undergoing coronary angiography (CAG). A total of 1,100 patients were initially screened. After applying inclusion and exclusion criteria, 550 patients were ultimately enrolled. Among these, 59 patients (10.7%) developed contrast-induced acute kidney injury (CI-AKI) and 491 patients (89.3%) did not. Abbreviations: ACS, acute coronary syndrome; CI-AKI, contrast-induced acute kidney injury.

A total of 1,100 patients with ACS were screened in this study. Based on the inclusion and exclusion criteria, 550 patients were ultimately enrolled (the study flowchart is shown in Figure 1). Among them, 59 patients developed CI-AKI after the procedure, yielding an incidence of 10.7%. In the entire cohort, 391 patients (71.2%) were male. Compared with the non-CI-AKI group, patients in the CI-AKI group were older (*p* < 0.001), had higher prevalence rates of hypertension, diabetes mellitus, and CKD (*p* = 0.010, *p* < 0.001, and *p* = 0.002, respectively), had higher HbA1c levels (*p* < 0.001), had lower preoperative eGFR levels (*p* < 0.001), had a lower postoperative hydration rate (*p* < 0.001), received a larger volume of intraoperative contrast agent (*p* = 0.010), had a greater number of stents implanted (*p* = 0.020), and had a higher proportion of patients undergoing PCI (*p* = 0.034). In terms of TG2 levels, both preoperative and postoperative sTG2 and uTG2 levels were significantly higher in the CI-AKI group than in the non-CI-AKI group (all *p* < 0.05). Detailed baseline clinical characteristics and laboratory findings for all patients are presented in Table 1.

**Table 1.** The baseline characteristics of participants in non-CI-AKI and CI-AKI groups.

Variables	Non-CI-AKI (*n* = 491)	CI-AKI (*n* = 59)	*p*-value
Gender/Male (*n*, %)	383 (78.0%)	42 (71.2%)	0.238
Age (years)	62 ± 12	75 ± 8	<0.001
BMI (kg/m^2^)	23.8 ± 3.0	23.3 ± 2.9	0.233
Smoker (*n*, %)	245 (49.9%)	24 (40.7%)	0.181
Hypertension (*n*, %)	272 (55.4%)	43 (72.9%)	0.010
DM (*n*, %)	91 (18.5%)	25 (42.4%)	<0.001
CKD (*n*, %)	16 (3.3%)	7 (11.9%)	0.002
HbAlc (%)	5.9 ± 1.0	6.7 ± 1.1	<0.001
TG (mmol/L)	1.79 ± 1.41	1.53 ± 0.78	0.171
TC (mmol/L)	4.47 ± 1.04	4.39 ± 1.07	0.623
LDL (mmol/L)	2.79 ± 0.88	2.74 ± 0.90	0.620
HDL (mmol/L)	1.10 ± 0.27	1.13 ± 0.28	0.301
sCr (μmol/L)	79 ± 23	85 ± 29	0.205
BUN (mmol/L)	5.7 ± 2.1	6.4 ± 2.7	0.065
eGFR (ml/min)	86.0 ± 21.3	73.2 ± 20.8	<0.001
UA (μmol/L)	358 ± 100	345 ± 80	0.711
Pre-op-sTG2 (pg/ml)	142.66 ± 19.00	173.64 ± 24.61	<0.001
Post-op-sTG2 (pg/ml)	153.30 ± 26.15	274.76 ± 75.64	<0.001
Pre-op-uTG2 (pg/ml)	138.18 ± 17.39	144.57 ± 11.50	0.007
Post-op-uTG2 (pg/ml)	142.91 ± 26.54	178.05 ± 42.41	<0.001
Medications			
Aspirin (*n*, %)	478 (97.4%)	58 (98.3%)	0.661
Clopidogrel /Ticagrelor (*n*, %)	489 (99.6%)	59 (100.0%)	0.623
β-blocker (*n*, %)	365 (74.3%)	43 (72.9%)	0.809
ACEI/ARB (*n*, %)	246 (50.1%)	34 (57.6%)	0.275
Statin (*n*, %)	486 (99.0%)	58 (98.3%)	0.636
PCI (*n*, %)	416 (84.7%)	56 (94.9%)	0.034
Number of lesion vessels	2.9 ± 1.5	2.8 ± 1.4	0.552
Three-vessel disease (*n*, %)	238 (48.5%)	25 (42.4%)	0.376
Number of stents	1.0 ± 0.5	1.2 ± 0.5	0.020
Hydration (*n*, %)	471 (95.9%)	42 (71.2%)	<0.001
Dose of contrast agent (ml)	109 ± 28	118 ± 29	0.010

CI-AKI: Contrast-Induced Acute Kidney Injury; BMI: Body Mass Index; DM: Diabetes Mellitus (defined as fasting glucose ≥7.0 mmol/L: HbA1c ≥6.5%: or current antidiabetic medication use); CKD: Chronic Kidney Disease (defined as eGFR <60 mL/min/1.73 m² or evidence of kidney damage); HbA1c: Glycated Hemoglobin; TG: Triglycerides; TC: Total Cholesterol; LDL: Low-Density Lipoprotein; HDL: High-Density Lipoprotein; sCr: Serum Creatinine; BUN: Blood Urea Nitrogen; eGFR: Estimated Glomerular Filtration Rate; UA: Uric Acid; Pre-op-sTG2: Preoperative Serum Transglutaminase 2; Post-op-sTG2: Postoperative Serum Transglutaminase 2; Pre-op-uTG2: Preoperative Urinary Transglutaminase 2; Post-op-uTG2: Postoperative Urinary Transglutaminase 2; ACEI: Angiotensin-Converting Enzyme Inhibitor; ARB: Angiotensin II Receptor Blocker; PCI: Percutaneous Coronary Intervention. Smoker defined as current active cigarette smoking at the time of admission. Hydration defined as completion of the full prescribed hydration protocol.

### Correlation analysis between TG2 levels and CI-AKI occurrence

To investigate the relationship between dynamic changes in TG2 levels and the occurrence of CI-AKI, the differences between preoperative and postoperative sTG2 and uTG2 levels (ΔsTG2 = postoperative sTG2 − preoperative sTG2, ΔuTG2 = postoperative uTG2 − preoperative uTG2) were calculated for each patient. The results showed that both ΔsTG2 and ΔuTG2 were significantly higher in the CI-AKI group than in the non-CI-AKI group (both *p* < 0.001).

**Figure 2.**
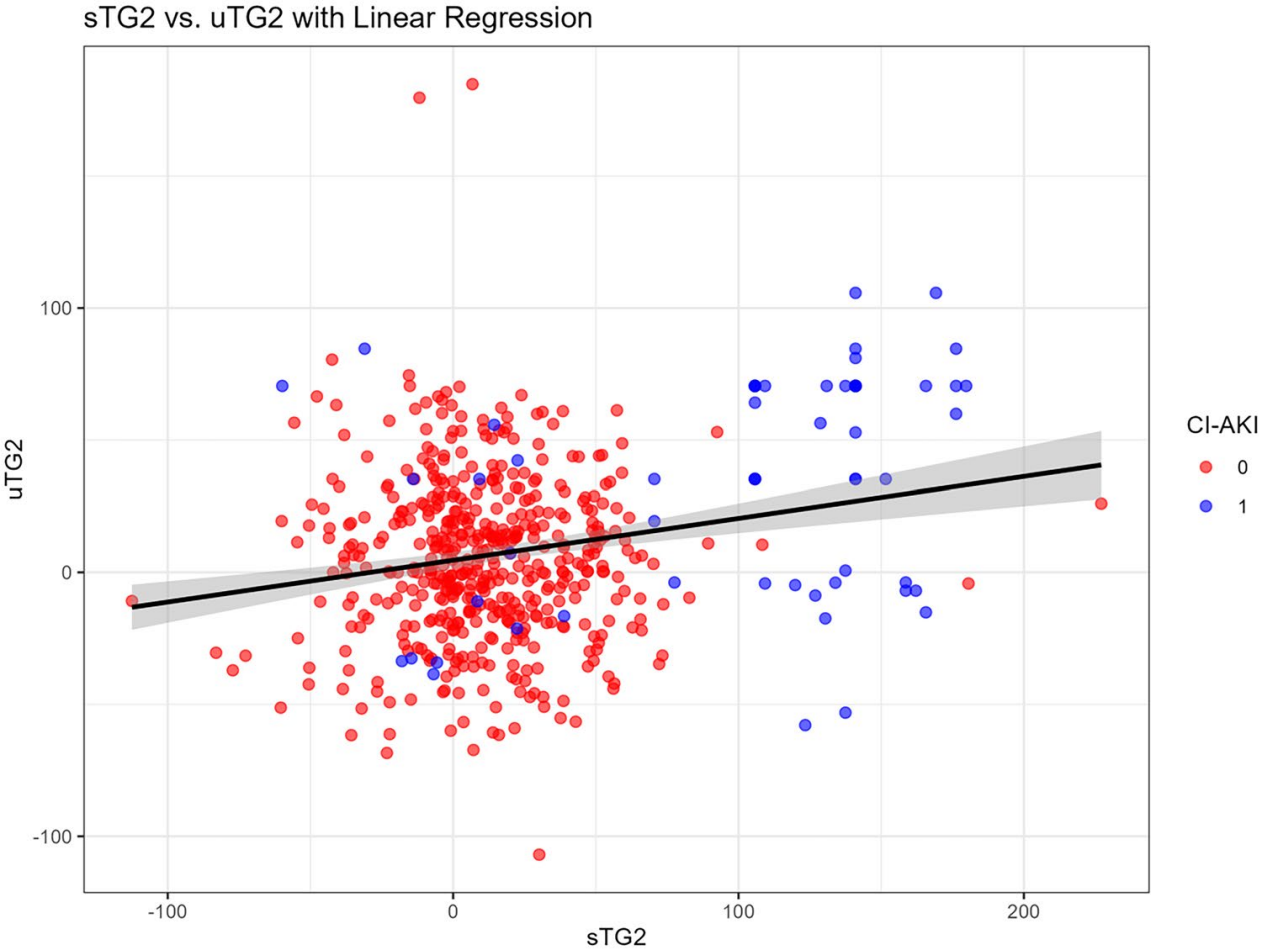
Correlation Between Peri-Procedural Changes in Serum and Urinary TG2 and CI-AKI Occurrence. Linear regression analysis showing the association between ΔsTG2 (difference in serum transglutaminase-2, postoperative minus preoperative, pg/mL) and ΔuTG2 (difference in urinary transglutaminase-2, postoperative minus preoperative, pg/mL) and the occurrence of CI-AKI. Each point represents an individual patient (blue: non-CI-AKI, red: CI-AKI). The solid line represents the regression line, and the shaded area represents the 95% confidence interval. Both ΔsTG2 (β = 0.36, 95% CI: 0.30–0.42, *p* < 0.001) and ΔuTG2 (β = 0.18, 95% CI: 0.11–0.25, *p* < 0.001) were significantly positively correlated with CI-AKI occurrence, indicating that greater peri-procedural increases in TG2 levels are associated with a higher risk of CI-AKI. R^2^ values are shown in each panel. Abbreviations: CI-AKI, contrast-induced acute kidney injury; ΔsTG2, difference in serum transglutaminase-2; ΔuTG2, difference in urinary transglutaminase-2.

Linear regression analysis revealed that increases in ΔsTG2 (β = 0.36, 95% *CI*: 0.30–0.42, *p* < 0.001) and ΔuTG2 (β = 0.18, 95% *CI*: 0.11–0.25, *p* < 0.001) were significantly positively correlated with the occurrence of CI-AKI (Figure 2), suggesting that dynamic elevation of TG2 levels is closely associated with the risk of CI-AKI.

### Construction and validation of the CI-AKI prediction model

**Figure 3.**
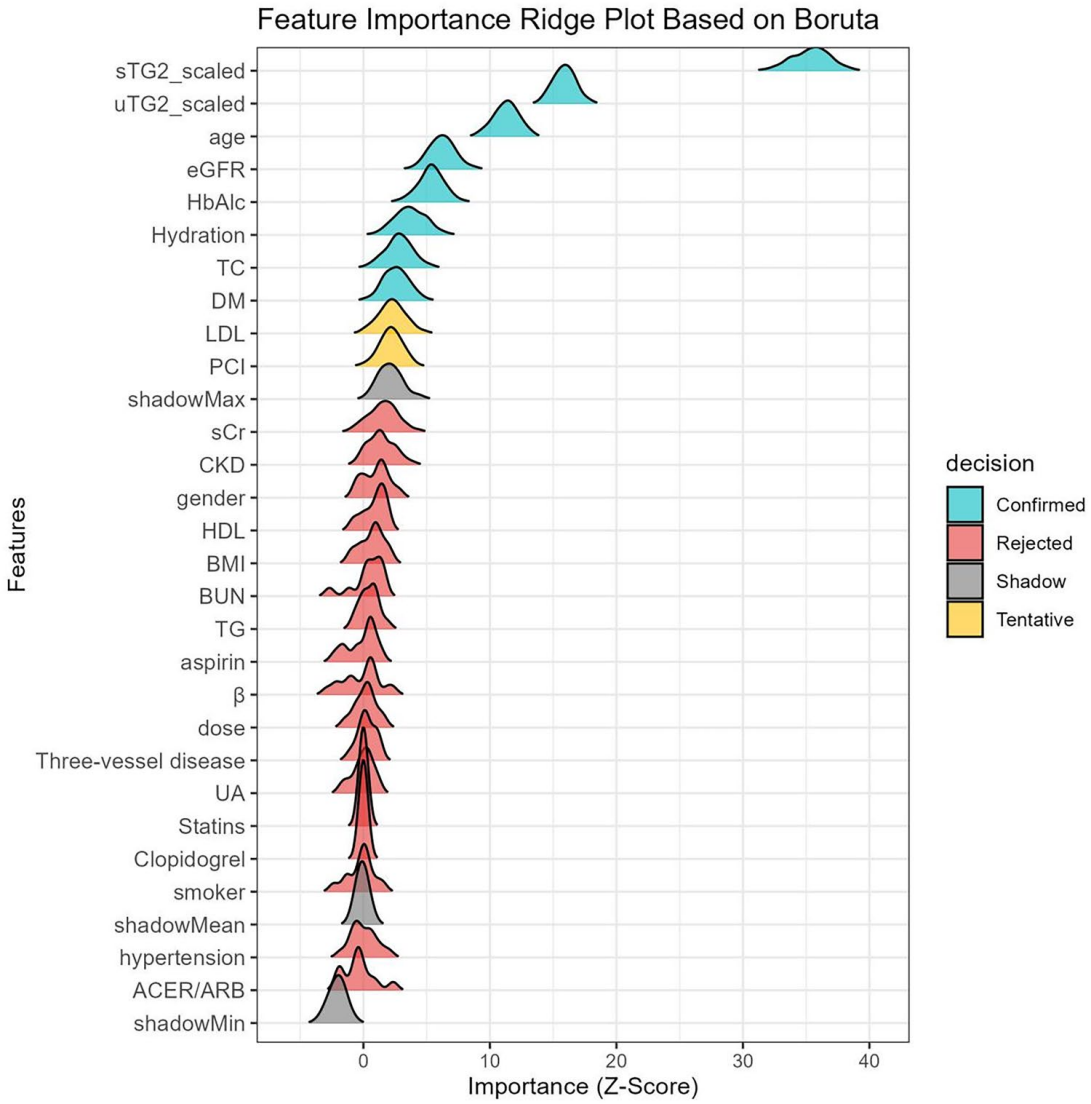
Variable Selection Using the Boruta Algorithm for CI-AKI Prediction. Importance ranking of clinical and biomarker variables for predicting CI-AKI using the Boruta algorithm. The y-axis shows the variable importance score (mean decrease in accuracy), and the x-axis lists the variables in descending order of importance. Variables are color-coded according to the Boruta algorithm classification: green boxes = confirmed important variables (age, HbA1c, total cholesterol [TC], diabetes mellitus [DM], estimated glomerular filtration rate [eGFR], postoperative hydration status, ΔsTG2, and ΔuTG2); yellow boxes = tentative variables (gender, BMI, hypertension, CKD, LDL, HDL, TG, smoking status); red boxes = unimportant variables (medication use variables, contrast volume, number of stents, number of diseased vessels, three-vessel disease, preoperative sCr, BUN, UA). The Boruta algorithm was run with 500 trees, and variables with a Z-score above the shadow variable maximum were considered confirmed. Variables identified as important by Boruta were subsequently entered into Lasso regression for further variable selection. Abbreviations: CI-AKI, contrast-induced acute kidney injury; BMI, body mass index; DM, diabetes mellitus; CKD, chronic kidney disease; HbA1c, glycated hemoglobin; TG, triglycerides; TC, total cholesterol; LDL, low-density lipoprotein; HDL, high-density lipoprotein; sCr, serum creatinine; BUN, blood urea nitrogen; eGFR, estimated glomerular filtration rate; UA, uric acid; Pre-op, preoperative; Post-op, postoperative; sTG2, serum transglutaminase-2; uTG2, urinary transglutaminase-2; ΔsTG2, difference in serum TG2; ΔuTG2, difference in urinary TG2; ACEI, angiotensin-converting enzyme inhibitor; ARB, angiotensin II receptor blocker.

All 550 patients were randomly divided into a training set (*n* = 385) and a test set (*n* = 165) at a ratio of 7:3 for model development and validation. The Boruta algorithm was first used to screen for characteristic variables significantly affecting the occurrence of CI-AKI. The results indicated that variables such as age, HbA1c, TC, history of diabetes mellitus, eGFR, postoperative hydration status, ΔsTG2, and ΔuTG2 had a significant impact on the occurrence of CI-AKI (Figure 3).

Based on the Boruta algorithm results, Lasso regression was further applied for variable selection. The regression coefficients for eGFR and LDL-C were compressed to zero, suggesting a minimal contribution to the predictive performance of the model; thus, these variables were excluded. The variables ultimately included in the multivariable logistic regression model were age, history of diabetes mellitus, HbA1c, TC, PCI treatment, postoperative hydration, ΔsTG2, and ΔuTG2. Multivariable logistic regression analysis showed that ΔsTG2 (OR = 4.019, 95% *CI*: 2.851–11.118, *p* < 0.001) and ΔuTG2 (OR = 1.396, 95% *CI*: 0.989–3.082, *p* < 0.001) were independent risk factors for CI-AKI. In addition, age (OR = 1.098, *p* < 0.001), history of DM (OR = 2.605, *p* = 0.005), HbA1c (OR = 1.192, *p* < 0.001), TC (OR = 1.684, *p* < 0.001), and PCI treatment (OR = 4.365, *p* < 0.001) were also independent risk factors for CI-AKI, while postoperative hydration (OR = 0.214, *p* < 0.001) was an independent protective factor (Table 2). Multicollinearity assessment using VIF showed that all variables in the final model had VIF values below 2 (range: 1.29–1.82), indicating no significant collinearity between predictors, including ΔsTG2 and ΔuTG2 (VIF: 1.82 and 1.76, respectively; Table S1).

**Table 2.** Multivariable logistic regression analysis of independent risk factors for CI-AKI.

Variables	Coefficient	OR value	95%CI	*p*-value
Age	0.094	1.098	1.047–1.185	<0.001
DM	0.957	2.605	1.000–5.091	<0.001
HbAlc	0.176	1.192	0.813–1.586	<0.001
Hydration	−1.544	0.214	0.032–1.000	<0.001
eGFR	0	–	–	–
TC	0.521	1.684	1.000–5.412	<0.001
LDL	0	–	–	–
PCI	1.474	4.365	1.000–6.918	<0.001
sTG2	1.391	4.019	2.851–11.118	<0.001
uTG2	0.333	1.396	0.989–3.082	<0.001

DM: Diabetes Mellitus; HbAlc: Glycated Hemoglobin; eGFR: Estimated Glomerular Filtration Rate; TC: Total Cholesterol; LDL: Low-Density Lipoprotein; PCI: Percutaneous Coronary Intervention; sTG2: Serum Transglutaminase 2; uTG2: Urinary Transglutaminase 2; OR: Odds Ratio; CI: Confidence Interval.

**Figure 4.**
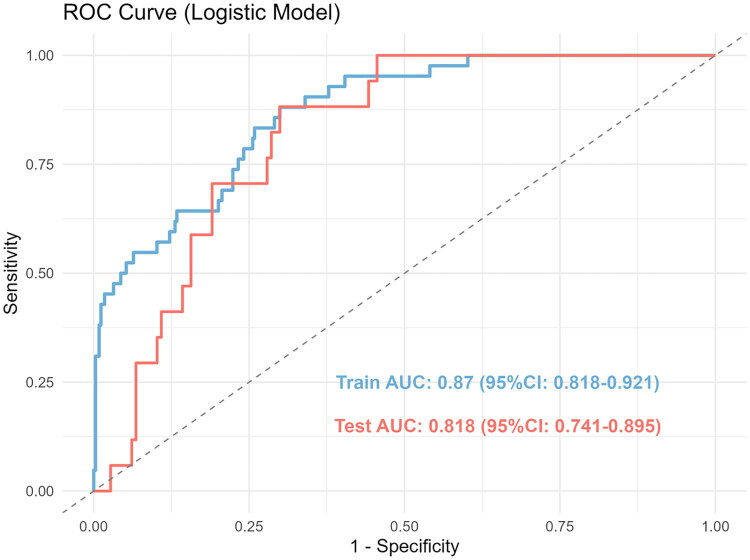
Receiver Operating Characteristic (ROC) Curves for the CI-AKI Prediction Model. ROC curves of the multivariable logistic regression model for predicting CI-AKI in (A) the training set (*n* = 385) and (B) the test set (*n* = 165). The model incorporated eight variables: age, diabetes mellitus, HbA1c, total cholesterol, PCI treatment, hydration completion, ΔsTG2, and ΔuTG2. The area under the curve (AUC) values, optimal cut-off points, sensitivity, and specificity are shown in each panel. In the training set, the model achieved an AUC of 0.870 (95% CI: 0.818–0.921), with an optimal cut-off value of 0.072, sensitivity of 88.1%, and specificity of 70.1%. In the test set, the model achieved an AUC of 0.818 (95% CI: 0.741–0.895), with an optimal cut-off value of 0.070, sensitivity of 88.2%, and specificity of 70.1%. The diagonal dashed line represents the reference line (AUC = 0.5, no discriminative ability). The gray shaded area represents the 95% confidence interval of the ROC curve. These results demonstrate good to excellent discriminative ability of the model in both internal validation cohorts. Abbreviations: CI-AKI, contrast-induced acute kidney injury; AUC, area under the curve; CI, confidence interval; ΔsTG2, difference in serum transglutaminase-2; ΔuTG2, difference in urinary transglutaminase-2; PCI, percutaneous coronary intervention.

**Figure 5.**
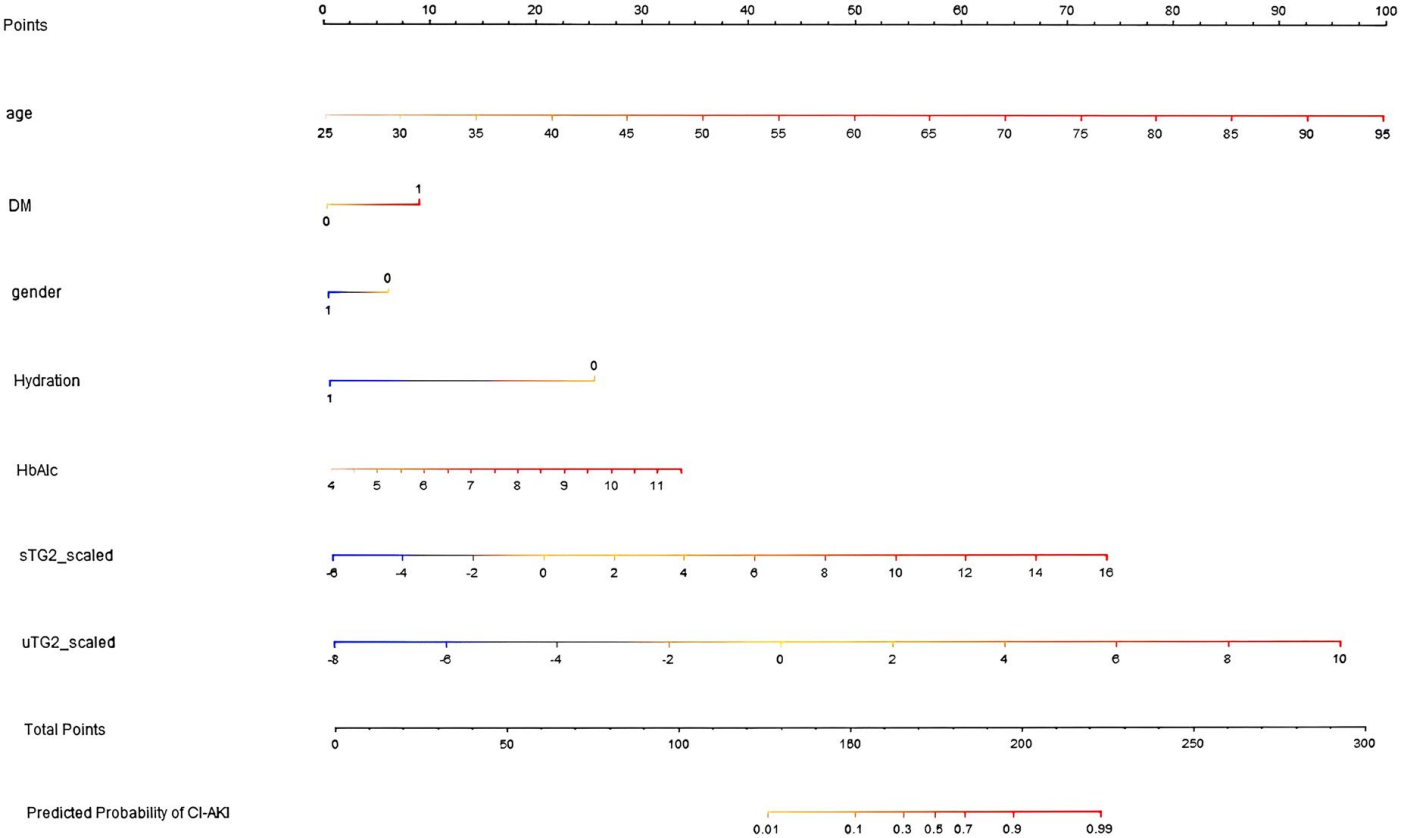
Nomogram for Predicting CI-AKI Risk in ACS Patients Undergoing Coronary Angiography. This nomogram incorporates seven clinical and biomarker variables to predict the individual probability of CI-AKI: ΔsTG2 (difference in serum TG2, pg/mL), age (years), diabetes mellitus (yes/no), total cholesterol (TC, mmol/L), HbA1c (%), ΔuTG2 (difference in urinary TG2, pg/mL), and hydration completion (yes/no). Each variable is assigned a score on the ‘Points’ scale. The sum of all individual scores yields the ‘Total Points’ (range 0–300), which corresponds to the predicted probability of CI-AKI on the ‘Risk Probability’ scale. Color coding: Variables are color-coded to facilitate interpretation: red/orange gradients indicate risk-increasing factors (higher values correspond to higher points); green indicates the protective factor.

A multivariable logistic regression prediction model was constructed based on the aforementioned eight variables. ROC curve analysis demonstrated that the model achieved an AUC of 0.870 (95%*CI*: 0.818–0.921) in the training set, with an optimal cut-off value of 0.072, corresponding to a sensitivity of 88.1% and a specificity of 70.1%. In the test set, the model achieved an AUC of 0.818 (95%*CI*: 0.741–0.895), with an optimal cut-off value of 0.070, corresponding to a sensitivity of 88.2% and a specificity of 70.1% (Figure 4), indicating good discriminative ability. The calibration curve showed good agreement between the predicted probability of CI-AKI and the actual observed probability (Hosmer–Lemeshow test, *p* = 0.362). The DCA demonstrated that the prediction model provided positive net benefit across threshold probabilities of 0.07–0.11 (Figure S1). The model consistently outperformed both the ‘treat all’ and ‘treat none’ strategies across clinically relevant threshold ranges, indicating that using this model to guide CI-AKI prevention strategies would provide net clinical benefit. These findings suggest that the model has practical utility for clinical decision-making, particularly for identifying high-risk patients who may benefit from intensified preventive measures. A nomogram prediction model incorporating the seven variables was constructed based on the regression coefficients from the Lasso analysis (Figure 5). This nomogram enables individualized risk assessment for postoperative CI-AKI in patients with ACS by quantifying the contribution of each variable.

## Discussion

The present study makes several important contributions to the field of CI-AKI prediction and prevention. First, to our knowledge, this is the first study to demonstrate a significant association between peri-procedural changes in serum and urinary TG2 and the occurrence of CI-AKI in patients with ACS undergoing coronary angiography. We found that patients who developed CI-AKI had substantially higher preoperative and postoperative TG2 levels, and—more importantly—greater peri-procedural increases in both serum and urinary TG2 (ΔsTG2 and ΔuTG2) compared to those who did not. These dynamic changes were independent risk factors for CI-AKI and demonstrated good predictive performance. Second, we developed and internally validated a novel predictive model incorporating ΔsTG2, ΔuTG2, and readily available clinical variables (age, diabetes status, HbA1c, total cholesterol, PCI treatment, and hydration status). The model’s AUC of 0.870 in the training set and 0.818 in the test set indicates excellent discriminatory ability, suggesting that TG2 adds incremental predictive value beyond conventional risk factors. The corresponding nomogram provides an intuitive tool for individualized risk assessment that could be readily implemented in clinical practice. Third, our findings provide new insights into the potential pathophysiology of CI-AKI. The observation that TG2 levels rise significantly in patients who develop CI-AKI supports the hypothesis that TG2 may participate in key pathological processes, including renal tissue hypoxia, calcium overload, inflammation, and tubular cell apoptosis—mechanisms we elaborate on in subsequent paragraphs. Importantly, the elevation of both serum and urinary TG2 suggests that TG2 reflects both systemic and local renal responses to contrast media exposure. In our cohort of 550 ACS patients, the overall CI-AKI incidence was 10.7%, which is consistent with previous reports from both domestic and international studies and comparable to rates reported in other high-risk populations (3.6%–18.3%) [23–25]. In addition to TG2, we identified advanced age, diabetes mellitus, CKD, low preoperative eGFR, high contrast volume, PCI, and low postoperative hydration rate as risk factors for CI-AKI, findings that align with established literature [25] and support the validity of our baseline data. Taken together, these findings position TG2 as a promising novel biomarker for CI-AKI risk stratification and provide a foundation for future mechanistic studies and potential therapeutic interventions targeting TG2-mediated pathways.

The clinical significance of our findings must be considered in the context of the diverse ACS population. While our cohort included patients with both unstable angina and NSTEMI, as well as STEMI patients presenting beyond the reperfusion time window, we excluded emergency primary PCI patients—a population known to have the highest CI-AKI risk. Studies specifically focusing on STEMI patients undergoing primary PCI have reported CI-AKI incidence rates ranging from 10% to over 20% [23,26,27]. This elevated risk reflects the unique pathophysiological challenges in STEMI: acute myocardial ischemia triggers systemic inflammation and oxidative stress; hemodynamic instability may compromise renal perfusion; and the urgent nature of the procedure often precludes optimal preoperative preparation, including adequate hydration [28]. Notably, the incidence of CI-AKI in STEMI patients appears to be influenced by multiple procedural and patient-related factors, including ischemic time, infarct size, hemodynamic status, and baseline renal function [26]. These observations underscore the critical need for reliable predictive biomarkers that can be rapidly assessed in the emergency setting to guide preventive strategies. Whether TG2 retains its predictive value in this highest-risk emergency population remains an open question that warrants dedicated investigation. The exclusion of emergency cases in our study represents a deliberate design choice to ensure protocol standardization and data completeness, but it also highlights an important gap that future studies should address.

The key innovation of this study lies in its initial exploration of the expression changes and predictive value of TG2 in CI-AKI among patients with ACS. The currently understood pathogenesis of CI-AKI is complex. Direct cytotoxicity of contrast agents can lead to injury of renal tubular epithelial cells and endothelial cells [29]; mitochondrial dysfunction in injured cells further induces apoptosis or necrosis and activates inflammatory responses. Concurrently, contrast agents can disrupt sodium transport in the renal tubules, leading to renal vasoconstriction, decreased renal blood flow, and exacerbated medullary ischemia and hypoxia [30]. Moreover, contrast agents may exacerbate renal tissue hypoxia by increasing the release of vasoconstrictive substances, reducing vasodilator levels, elevating blood viscosity, and decreasing the oxygen-carrying capacity of red blood cells [31]. Oxidative stress resulting from increased reactive oxygen species generation can induce intracellular lipid peroxidation, protein denaturation, and DNA damage, further amplifying renal injury [32]. Recent studies have also indicated that iodide ions in contrast agents can trigger nuclear factor-κB-mediated immune-inflammatory responses, promoting cytokine release and inflammatory cell infiltration, thereby exacerbating endothelial injury in the kidney [33].

As a key stress-responsive protein, TG2 is significantly activated under various pathological conditions, including hypoxia, inflammation, and oxidative stress. It may contribute to the development and progression of CI-AKI through the following mechanisms: First, under hypoxic conditions in renal tissue, TG2 activity in red blood cells is markedly increased, promoting the production of 2,3-diphosphoglycerate, which facilitates oxygen release from hemoglobin to alleviate tissue hypoxia [34]. However, during CI-AKI, the direct toxic effects of contrast agents on red blood cells, along with hemoconcentration and decreased erythrocyte deformability, can lead to extensive red blood cell damage, potentially attenuating the compensatory hypoxic response mediated by TG2. This may delay the alleviation of renal tissue hypoxia and thereby promote the occurrence of CI-AKI. Second, the conformational state of TG2 is regulated by local Ca^2+^ concentration, GTP/GDP levels, and substrate availability, existing in either an open or closed conformation. Most of its biological functions require the open conformation [35]. Intracellular calcium overload is a key pathological event in CI-AKI [36]; in the high-calcium environment induced by contrast agents, TG2 may tend to remain in its open conformation, thereby promoting renal vasoconstriction and exacerbating renal ischemia and hypoxia. Third, TG2 can function as a nitrosylase, regulating glycolysis, redox balance, and inflammatory responses during endothelial cell injury. TG2-mediated reduction in nitric oxide bioavailability may further aggravate renal vasoconstriction and ischemia [37]. Fourth, with regard to inflammation, Shinoda et al. demonstrated that TG2 expression levels increase in parallel with M2 macrophage markers, and that TG2 knockout or inhibition significantly suppresses M2 macrophage polarization [38], suggesting that TG2 may participate in inflammatory cell infiltration in CI-AKI by modulating macrophage polarization, thereby exacerbating renal injury. Fifth, in terms of apoptosis, Piacentini et al. found that TG2 overexpression promotes apoptosis by increasing mitochondrial membrane potential and cellular oxidative stress levels [39]; moreover, when mitochondrial integrity is compromised, TG2 can be recruited to damaged mitochondria, further accelerating apoptosis [40]. Since renal tubular cell apoptosis is a central pathological feature of CI-AKI, TG2 may contribute to the pathogenesis of CI-AKI by promoting renal tubular cell apoptosis. These mechanisms are interrelated and synergistic, collectively forming a potential pathological network through which TG2 may be involved in CI-AKI. However, the specific molecular mechanisms require further validation through basic experimental studies. We acknowledge that our current study did not include direct measurements of inflammatory markers or extracellular matrix remodeling indicators, which limits our ability to directly validate the proposed inflammatory and fibrotic pathways. As a clinical observational study focused on the predictive value of TG2 for CI-AKI onset, we prioritized the measurement of TG2 levels and standard clinical parameters within the constraints of specimen availability and budgetary limitations. Future studies incorporating comprehensive inflammatory biomarker panels and extracellular matrix components would be valuable to determine whether the observed association between TG2 and CI-AKI is mediated through these pathways. Such investigations could potentially establish TG2 not only as a predictive biomarker but also as a therapeutic target for preventing CI-AKI and its long-term fibrotic sequelae.

The strengths of this study include its prospective cohort design, which strictly adhered to the STROBE guidelines, thereby reducing selection bias inherent to retrospective studies. The simultaneous measurement of TG2 levels in both serum and urine, along with analysis of dynamic changes in relation to CI-AKI, provides a more comprehensive assessment than single-specimen testing. The combination of the Boruta algorithm with Lasso regression effectively eliminated redundant variables, enhancing the parsimony and practicality of the prediction model. The logistic regression model and nomogram offer an intuitive and convenient tool for individualized clinical risk assessment. The DCA further supports the clinical utility of our model, demonstrating that using the model to guide preventive interventions would provide net benefit across clinically relevant risk thresholds. This is particularly important for CI-AKI, where preventive measures such as hydration, contrast volume reduction, and pharmacological prophylaxis have demonstrated efficacy but carry some burden and cost. The model’s ability to identify high-risk patients can help clinicians target these interventions to those most likely to benefit, optimizing resource utilization and minimizing unnecessary interventions in low-risk patients.

This study has several limitations. First, it is a single-center study with a relatively small sample size, particularly the CI-AKI group comprising only 59 cases, which may lead to insufficient statistical power for multivariable analyses and subgroup analyses, potentially affecting the stability of the results. To mitigate this, we employed Lasso regression with 10-fold cross-validation for variable selection and internally validated the model using a train/test split. Nevertheless, the limited number of events precluded extensive subgroup analyses, and the results should be interpreted with appropriate caution. Larger multicenter studies with more CI-AKI events are needed to confirm our findings and enable robust subgroup analyses. Second, the single-center design may introduce selection bias, and the external validity of the findings requires confirmation in multicenter, large-scale cohort studies. Third, patients undergoing emergency PCI were excluded from this study. This population is known to have a higher incidence of CI-AKI [24] and represents a high-risk group where TG2 measurement might be particularly valuable. While STEMI patients presenting beyond the reperfusion time window and those who received thrombolytic therapy with delayed catheterization were potentially eligible, the exclusion of emergency cases may limit the generalizability of our findings to the full spectrum of ACS patients, particularly those at highest risk. Future studies specifically targeting emergency PCI populations are needed to determine whether TG2 retains its predictive value in this high-risk context. Fourth, the timing of postoperative TG2 specimen collection (within 72 h) was based on the diagnostic time point for CI-AKI, but there is no preliminary experimental evidence supporting this as the optimal time window for TG2 measurement. Future time-series studies are needed to characterize the dynamic changes in TG2 and identify the optimal sampling time. Furthermore, given the well-established role of TG2 in CKD progression and renal fibrosis [17,18], our findings raise the important question of whether peri-procedural TG2 elevations not only predict CI-AKI onset but also influence long-term renal outcomes, including the development or acceleration of CKD and fibrosis. We acknowledge that investigating the relationship between TG2 and the prognosis of CI-AKI—particularly long-term renal function decline, progression to CKD, and development of renal fibrosis—represents a clinically meaningful direction that warrants dedicated prospective studies with extended follow-up. Such studies would be instrumental in determining whether TG2 could serve as both an acute predictive biomarker and a prognostic indicator for long-term renal outcomes in patients with ACS undergoing coronary angiography. Fifth, the current high cost of TG2 testing and its absence from routine clinical laboratory panels limit its immediate clinical applicability. Sixth, the prediction model developed in this study was validated only in internal cohorts (training and test sets) and has not been externally validated in an independent cohort; its generalizability warrants further investigation. Seventh, as an observational study, this study can only establish an association between TG2 and CI-AKI, not causality. The specific molecular pathways—including inflammatory responses, renal tubular apoptosis, and extracellular matrix accumulation—through which TG2 may contribute to CI-AKI pathogenesis require further elucidation through basic research, including cellular and animal experiments. In particular, we did not measure inflammatory cytokines or fibrosis markers, which precludes direct validation of these proposed mechanistic pathways in our cohort. Future studies should incorporate such markers to clarify whether TG2’s association with CI-AKI is mediated through inflammatory and fibrotic mechanisms.

## Conclusion

In conclusion, this study demonstrates that peri-procedural increases in serum and urinary TG2 (ΔsTG2 and ΔuTG2) are independent risk factors for CI-AKI in ACS patients undergoing coronary angiography and provide incremental predictive value beyond conventional clinical risk factors. The predictive model incorporating these biomarkers achieves excellent discriminative performance (AUC 0.870), and the accompanying nomogram offers an intuitive tool for individualized risk assessment. These findings have direct translational relevance. CI-AKI remains a common and costly complication, affecting approximately 10% of ACS patients undergoing coronary procedures, and is associated with prolonged hospital stays, increased cardiovascular events, and elevated mortality. Current risk stratification relies primarily on clinical variables that have limited predictive accuracy. The addition of TG2 measurement—a simple blood and urine test—could substantially improve risk identification. Before clinical implementation, prospective validation in multicenter cohorts, particularly in emergency PCI populations, is essential. Additionally, development of rapid point-of-care TG2 assays would facilitate real-time clinical decision-making. Cost-effectiveness analyses and integration with existing risk scores should also be evaluated. Nevertheless, this study provides a strong foundation for TG2 as a novel biomarker that could meaningfully improve CI-AKI risk prediction and ultimately patient outcomes.

## Supplementary Material

Supplementary Table S1.docx

Figure S1.tif

## Data Availability

The original contributions presented in the study are included in the article/Supplementary material, further inquiries can be directed to the corresponding author.

## Abbreviations

BMI: body mass index
CI-AKI: contrast-induced acute kidney injury
DM: diabetes mellitus
CKD: chronic kidney disease
ACS: acute coronary syndrome
PCI: percutaneous coronary intervention
CM: contrast medium; CAG: coronary angiography
TGase: transglutaminase
AKI: acute kidney injury
TG2: transglutaminase-2
KDIGO: the Kidney Disease Improving Global Guidelines
ESUR: the European Society of Urogenital Radiology
sCr: serum creatinine
HbAlc: glycosylated hemoglobin
TG: triglycerides; TC: total cholesterol
LDL: low-density lipoprotein
HDL: high-density lipoprotein
BUN: lood urea nitrogen
eGFR: estimated glomerular filtration rate
UA: uric acid
ROC: receiver operating characteristic
ELISA: Enzyme-linked immunosorbent assay
