# Supplementary material for: Association of transglutaminase-2 with contrast-induced acute kidney injury in Chinese patients with acute coronary syndrome: a prospective cohort study
Source: Ann Med. 2026 Aug 10;58(1):2712061. doi: 10.1080/07853890.2026.2712061 (PMC13459302; doi:10.1080/07853890.2026.2712061)
Supplement: Supplementary file 1 [file IANN_A_2712061_SM9739.docx]

Supplementary Table S1. Variance Inflation Factor (VIF) for Variables in the Multivariable Logistic Regression Model

| Variables | VIF | Tolerance (1/VIF) |
| --- | --- | --- |
| ΔsTG2 | 1.82 | 0.549 |
| ΔuTG2 | 1.76 | 0.568 |
| Age | 1.45 | 0.690 |
| DM | 1.38 | 0.725 |
| HbA1c | 1.52 | 0.658 |
| TC | 1.33 | 0.752 |
| PCI | 1.41 | 0.709 |
| Hydration | 1.29 | 0.775 |
